# Correlations of the *CNR1* Gene with Personality Traits in Women with Alcohol Use Disorder

**DOI:** 10.3390/ijms25105174

**Published:** 2024-05-09

**Authors:** Filip Maciocha, Aleksandra Suchanecka, Krzysztof Chmielowiec, Jolanta Chmielowiec, Andrzej Ciechanowicz, Agnieszka Boroń

**Affiliations:** 1 Department of Clinical and Molecular Biochemistry, Pomeranian Medical University in Szczecin, Powstańców Wielkopolskich 72 St., 70-111 Szczecin, Poland; 83603@student.pum.edu.pl (F.M.); andrzej.ciechanowicz@pum.edu.pl (A.C.); 2 Independent Laboratory of Behavioral Genetics and Epigenetics, Pomeranian Medical University in Szczecin, Powstańców Wielkopolskich 72 St., 70-111 Szczecin, Poland; aleksandra.suchanecka@pum.edu.pl; 3 Department of Hygiene and Epidemiology, Collegium Medicum, University of Zielona Góra, 28 Zyty St., 65-046 Zielona Góra, Poland; chmiele@vp.pl (K.C.); chmiele1@o2.pl (J.C.)

**Keywords:** AUD, gene, *CNR1*, microsatellite, (AAT)n, personality

## Abstract

Alcohol use disorder (AUD) is a significant issue affecting women, with severe consequences for society, the economy, and most importantly, health. Both personality and alcohol use disorders are phenotypically very complex, and elucidating their shared heritability is a challenge for medical genetics. Therefore, our study investigated the correlations between the microsatellite polymorphism (AAT)n of the Cannabinoid Receptor 1 (*CNR1*) gene and personality traits in women with AUD. The study group included 187 female subjects. Of these, 93 were diagnosed with alcohol use disorder, and 94 were controls. Repeat length polymorphism of microsatellite regions (AAT)n in the *CNR1* gene was identified with PCR. All participants were assessed with the Mini-International Neuropsychiatric Interview and completed the NEO Five-Factor and State-Trait Anxiety Inventories. In the group of AUD subjects, significantly fewer (AAT)n repeats were present when compared with controls (*p* = 0.0380). While comparing the alcohol use disorder subjects (AUD) and the controls, we observed significantly higher scores on the STAI trait (*p* < 0.00001) and state scales (*p* = 0.0001) and on the NEO Five-Factor Inventory Neuroticism (*p* < 0.00001) and Openness (*p* = 0.0237; insignificant after Bonferroni correction) scales. Significantly lower results were obtained on the NEO-FFI Extraversion (*p* = 0.00003), Agreeability (*p* < 0.00001) and Conscientiousness (*p* < 0.00001) scales by the AUD subjects when compared to controls. There was no statistically significant Pearson’s linear correlation between the number of (AAT)n repeats in the *CNR1* gene and the STAI and NEO Five-Factor Inventory scores in the group of AUD subjects. In contrast, Pearson’s linear correlation analysis in controls showed a positive correlation between the number of the (AAT)n repeats and the STAI state scale (r = 0.184; *p* = 0.011; insignificant after Bonferroni correction) and a negative correlation with the NEO-FFI Openness scale (r = −0.241; *p* = 0.001). Interestingly, our study provided data on two separate complex issues, i.e., (1) the association of (AAT)n *CNR1* repeats with the AUD in females; (2) the correlation of (AAT)n *CNR1* repeats with anxiety as a state and Openness in non-alcohol dependent subjects. In conclusion, our study provided a plethora of valuable data for improving our understanding of alcohol use disorder and anxiety.

## 1. Introduction

Alcohol use disorder (AUD) is a significant issue affecting women, with severe consequences for society, the economy, and most importantly, health [1]. Alcohol use disorder is associated with a lower quality of life [2] and a higher risk of medical conditions such as cirrhosis [3], diabetes, and cancer [4]. Regular drinking increases the risk of breast cancer and alcohol-related heart disease. Drinking during pregnancy can result in low birth weight, premature birth, and lower APGAR scores [5]. Prenatal alcohol exposure also affects cognitive function, verbal learning, and spatial memory performance, causing widespread damage that affects the brain [6]. There is evidence that links AUD to depressive disorders [7]. Negative consequences of AUD include damage and inflammation in the brain [5], which is the most critical organ in the human body.

Studies involving human neuroimaging have revealed notable differences between sexes regarding brain functions, neural structure, and neurochemistry. This highlights the importance of considering sex as a modulating factor in the interpretation of brain-related data. Additionally, differences at the cellular level have also been observed, with men and women differing in cell type and receptor density [8].

Personality traits have also been found to vary between the sexes. Females have been observed to show increased levels of Neuroticism and Harm avoidance, which are anxiety-related traits, compared to males [9,10]. On the other hand, males tend to exhibit higher levels of persistence, openness to experience, self-confidence, and self-esteem [9,11]. Females, in comparison, demonstrate higher levels of conscientiousness, reward dependence, self-transcendence, cooperation and lower levels of self-directedness [10,12].

Robert Cloninger introduced the division of alcoholism into two types: type I, which more often affects women, and type II, which is more common in men. Type I and II alcoholism is characterised by personality traits like harm avoidance, novelty seeking, and reward dependence. In type I alcoholism, people show high harm avoidance, low novelty seeking, and high reward dependence. These personality traits correlate with high levels of anxiety, so that suggests these people drink to alleviate anxiety. Compared to type I, type II exhibits low harm avoidance, high novelty seeking, and low reward dependence, which indicate that drinking alcohol induces euphoria [13].

AUD research has frequently investigated the endocannabinoid system (ECS) function, a widely distributed neuromodulatory system associated with central nervous system development and synaptic plasticity [14,15,16]. It includes cannabinoid receptors (CB1 and CB2), endogenous cannabinoids, and the endocannabinoid degrading system (FAAH—fatty acid amide hydrolase). Cannabinoid receptors are negatively associated with adenylyl cyclase via the G protein [17]. CB1 receptors are mainly located in the brain—with high density in the cerebral cortex, hippocampus, cerebellum, striatum, and substantia nigra [18].

In 1992, Devane and his colleagues demonstrated that endogenous cannabinoids could bind to CB1 receptors in the mammalian brain [19]. These include arachidonylethanolamide (AEA), also known as anandamide, and 2-arachidonylglycerol (2-AG). Unlike classic neurotransmitters, AEA and 2-AG are not stored in synaptic vesicles but instead are secreted by neurons as needed. Changes to the endocannabinoid system at different levels within the brain may play a crucial role in the development of alcohol tolerance and dependence [20].

The *CNR1* gene is located on chromosome 6 (6q15), comprising eight exons and encoding 472 amino acids [21,22]. The regulation of *CNR1* expression is not yet fully understood [23]. One commonly reported alteration in this gene is microsatellite polymorphism (AAT)n, located at 18,000 base pairs 3′ to the gene [24,25]. This polymorphism has been investigated in various conditions, including schizophrenia [26], multiple sclerosis [27], Huntington’s Disease [28], Parkinson’s Disease [29], and addictions [30].

Patients diagnosed with multiple sclerosis (MS) who have a higher number of AAT repeats in their genes are at a greater risk of disease progression and may experience a more severe course of the disease. In addition, this genetic variation plays a crucial role in determining the severity of relapsing MS [27,31]. Research conducted by Kloster E et al. also suggests that there is a connection between the number of (AAT)n repeats and the age at which Huntington’s Disease (HD) develops [28].

Studies have shown that the (AAT)n polymorphism does not have a direct correlation with schizophrenia in the Chinese population [26]. On the other hand, research suggests that there is no correlation between the number of AAT repeats and susceptibility to depression [32]. However, for patients with Parkinson’s Disease (PD), those with more repeats (16 or more) have been found to be less prone to depression [29]. Additionally, (AAT)n and other polymorphisms of the *CNR1* gene are associated with addiction syndromes [30].

Despite numerous attempts to understand the role of polymorphism in alcohol addiction, research has yet to concentrate specifically on the female population. Thus, we have taken up the challenge of examining the correlation between personality traits and Alcohol Use Disorder (AUD) in women. AUD is a phenotypically complex disorder, with multiple genes playing a role. However, by utilising short tandem repeat polymorphisms, such as (AAT)n in the *CNR1* gene, we may partially explain the missing heritability of AUD in women.

The hypothesis put forth is that a greater number of AAT repeats in the 3′ region of the *CNR1* gene may result in a reduced availability of the CB1 receptor for endogenous cannabinoids, i.e., AEA and 2AG. This, in turn, may contribute to the occurrence of higher anxiety levels and, indirectly, to AUD in individuals who are predisposed to such conditions. The objective of this study is to verify the hypothesis by examining the following: (1) The association between the number of AAT repeats and the occurrence of AUD; (2) The anxiety levels of AUD and control subjects, measured as a state and as a trait, as well as the personality traits; (3) The correlation between the number of AAT repeats and anxiety levels measured as a state and as a trait, as well as the personality traits in AUD and control subjects.

Our study aims to fill the gap in current research by examining the correlations between personality traits and AUD in women. The results of this study will provide insight into the complex nature of AUD and inform future research in this field.

## 2. Results

**Figure 1:**
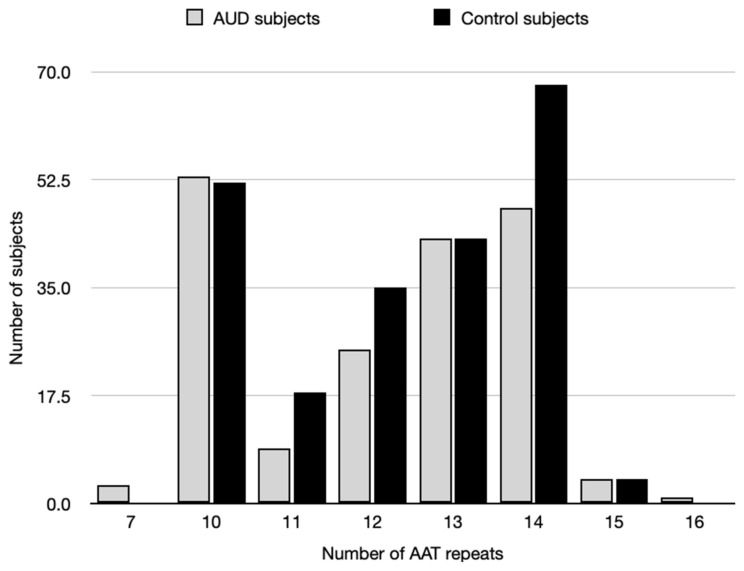
Alleles of (AAT)n polymorphism *CNR1* in the AUD and control subjects.

**Figure 2:**
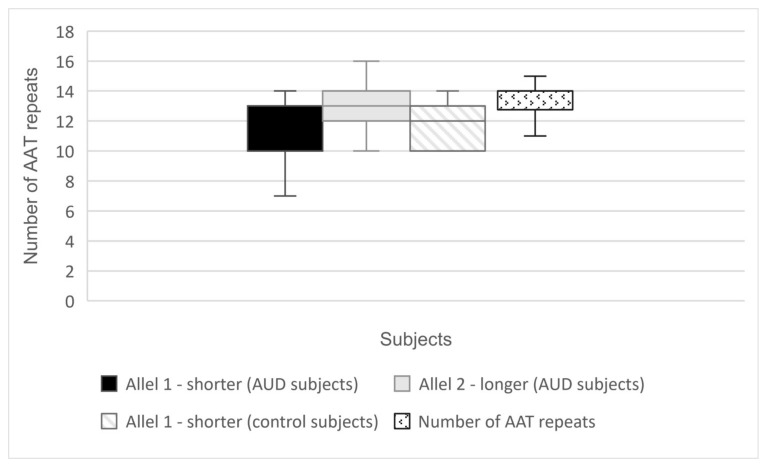
Distribution of (AAT)n repeats number in AUD and control subjects.

We found eight distinctive alleles in our study groups (AUD and control subjects) with a number of AAT repetitions ranging from 7 to 16 (Table 1; Figure 1 and Figure 2).

The analysis of the (AAT)n repeat number polymorphism in the *CNR1* gene revealed that in the group of alcohol use disorder subjects, significantly fewer repeats were present when compared with the controls (12.04 vs. 12.40, Z = −2.080, *p* = 0.0380, Table 2).

While comparing the alcohol use disorder subjects (AUD) and the controls, we observed significantly higher scores on the STAI trait (M 7.34 vs. M 4.92, *p* < 0.00001) and state scales (M 5.87 vs. M 4.43, *p* = 0.0001), and on the NEO Five-Factor Inventory Neuroticism (M 7.11 vs. M 4.46, *p* < 0.00001) and Openness (M 5.16 vs. M 4.42, *p* = 0.0237; insignificant after Bonferroni correction) scales. Significantly lower results were obtained on the NEO-FFI Extraversion (5.14 vs. 6.72, *p* = 0.00003), Agreeability (M 3.83 vs. M 5.52, *p* < 0.00001) and Conscientiousness (M 4.88 vs. M 7.05, *p* < 0.00001) scales by the AUD subjects when compared to controls (Table 3).

There was no statistically significant Pearson’s linear correlation between the number of (AAT)n repeats in the *CNR1* gene and the STAI scales and NEO Five-Factor Inventory traits studied in the group of AUD subjects. In contrast, Pearson’s linear correlation analysis in controls showed a positive correlation between the number of the (AAT)n repeats and the STAI state scale (r = 0.184; *p* = 0.011) and a negative correlation with the NEO-FFI Openness scale (r = −0.241; *p* = 0.001, Table 4).

**Figure 3:**
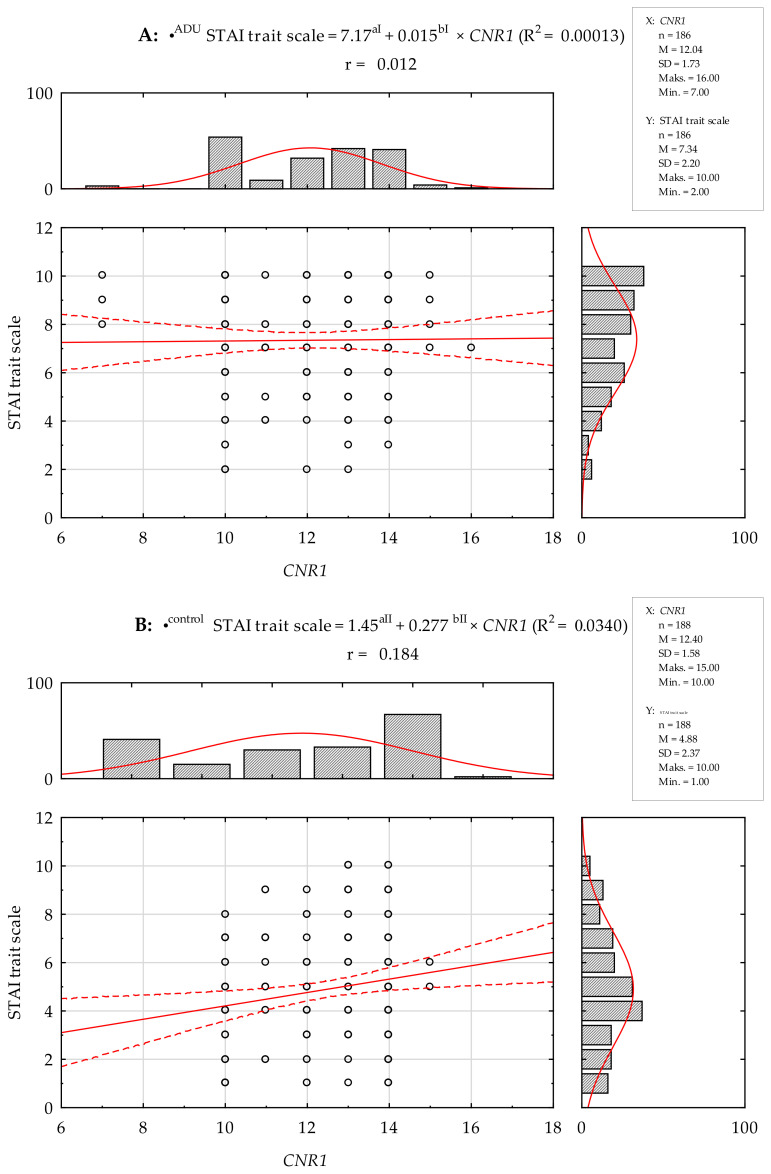
Pearson’s linear correlation between the number of (AAT)n repeats in the *CNR1* gene and STAI trait scale in a group of AUD (**A**) and control subjects (**B**). r—Pearson’s linear correlation. •^ADU^ regression linear models; R^2^ = 0.00013, ^aI^ *p* < 0.0001, ^bI^ *p* = 0.8745. •^control^ regression linear models; R^2^ = 0.0340, ^aII^ *p* = 0.2865, ^bII^ *p* = 0.0113. Red line—designated regression line, red dashed lines—95% Cl.

**Figure 4:**
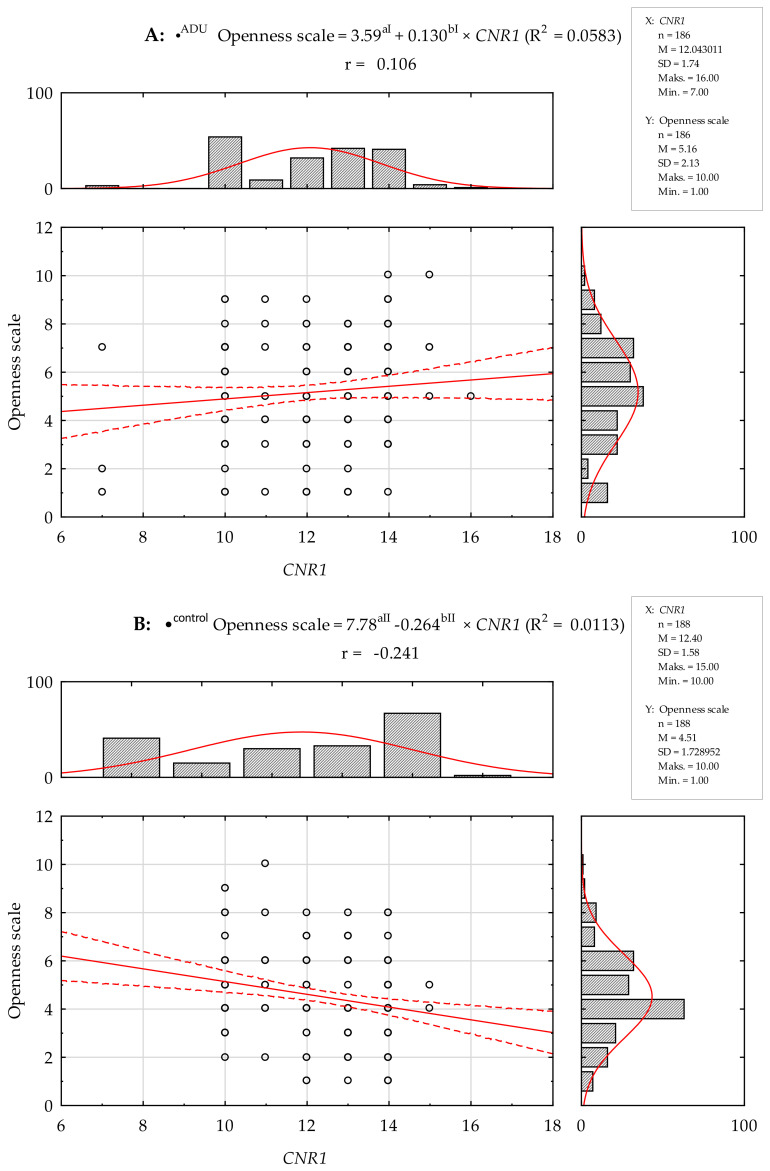
Pearson’s linear correlation between the number of (AAT)n repeats in the *CNR1* gene and NEO-FFI Openness scale in a group of AUD (**A**) and control subjects (**B**). r—Pearson’s linear correlation. •^ADU^ regression linear models; R^2^ = 0.0583, ^aI^ *p* = 0.0012, ^bI^ *p* = 0.0008. •^control^ regression linear models; R^2^ = 0.0113, ^aII^ *p* < 0.0001, ^bII^ *p* = 0.1492. Red line—designated regression line, red dashed lines—95% Cl.

Regression linear models were also determined between the number of (AAT)n repeats in the *CNR1* gene and the STAI scales and NEO Five-Factor Inventory traits studied in the group of AUD subjects. No statistically significant model was established for the STAI scales and NEO Five-Factor Inventory traits (Figure 3 and Figure 4). In the control group, a linear regression model was statistically determined for the STAI trait scale and Openness scale (Figure 3 and Figure 4).

## 3. Discussion

Researchers have been exploring the endocannabinoid system as a target for new anti-anxiety drugs. These drugs are designed to increase the availability of AEA and 2-AG or inhibit the enzymes FAAH and MAGL [33]. The hypothesis is that a greater number of AAT repeats in the 3′ region of the *CNR1* gene may result in a reduced availability of the CB1 receptor for endogenous cannabinoids, i.e., AEA and 2AG. This, in turn, may contribute to the occurrence of higher anxiety levels and indirectly to AUD in individuals who are predisposed to such conditions. The objective of this study was to verify the hypothesis by examining the following: (1) The association between the number of AAT repeats and the occurrence of AUD; (2) The anxiety levels of AUD and control subjects, measured as a state and as a trait, as well as the personality traits; and (3) The correlation between the number of AAT repeats and anxiety levels measured as a state and as a trait, as well as the personality traits in AUD and control subjects.

### 3.1. Cannabinoid System

The brain is a complex organ that is influenced by sex hormones during fetal development and throughout adulthood [34]. Various brain circuits show apparent differences between males and females, particularly in regions associated with stress, emotions, memory, and cognition [35]. One such system is the endogenous cannabinoid system, which regulates physiological processes. This system emerges early during brain development and is sexually dimorphic, meaning that male and female brains display different levels of endocannabinoids, different endocannabinoid-mediated glial cell genesis in the developing brain, and different cannabinoid subtype 1 receptor (CB1R) density and affinity [36,37,38].

Research has shown that the differences in the endocannabinoid system between males and females are significant and region-dependent. The density and functionality of CB1R vary between the two sexes, and sex affects the endocannabinoid system in many brain areas [39,40].

The distribution of neuronal CB1Rs in the brain is not uniform, with higher concentrations found in areas that regulate cognition and short-term memory (cerebral cortex and hippocampus) and motor functions (basal ganglia and cerebellum) [18,41,42].

The endocannabinoid system is susceptible to early life stress, and this sensitivity is dependent on sex and brain region. Changes in gene expression are evident in the adolescent brain [43].

Studies on humans have revealed that the number of CB1Rs increases in females but not males with ageing [44]. Women show an age-dependent increase in CB1Rs in the basal ganglia, lateral temporal cortex, and hippocampus. At the same time, men display enhanced binding in the limbic system and cortico-striato-thalamic-cortical circuit. Interestingly, adolescent cannabinoid exposure has long-term and sex-dependent effects on adult hippocampal neurogenesis and stress response [45].

Research has shown that cannabinoids affect the body and behaviour differently in males and females [46,47]. While males are more affected by cannabinoids’ impact on food intake and energy balance, females are more impacted by their effects on anxiety and depression [48,49]. Interestingly, female marijuana smokers experience weaker effects than their male counterparts in terms of delta9-THC levels [50] and their impact on cardiovascular and subjective experiences [51]. However, male non-marijuana smokers are more sensitive to the subjective effects of delta9-THC than women [52]. Additionally, female marijuana smokers are more likely to experience dizziness, hemodynamic changes, and visuospatial memory impairment than male smokers [53].

Studies have shown that in rodents, the effects of cannabinoids on catalepsy, antinociception, and changes in locomotor activity are more pronounced in females than in males [54]. Additionally, while females experience the effects of cannabinoids on exploratory behaviour and anxiety, males do not [55]. It has also been reported that gender-related sociocultural distinctions and anatomical and physiological differences between males and females can affect impulsive and compulsive behaviour as well as other dysregulated behaviours [56,57].

Differences in the rate of cell proliferation depending on sex were also demonstrated, with greater proliferation in the amygdala of female newborns [37]. Sex differences were also observed in the levels of endocannabinoids and their metabolic enzymes in the amygdala. The amygdala of males showed higher levels of 2-AG and anandamide, while the amygdala of females contained greater concentrations of their primary metabolic enzymes, MAGL and FAAH, respectively. These findings suggest that differences in endocannabinoid tone between sexes develop early and may lead to differential development of the system, potentially resulting in different responses to exogenous cannabinoids later on [58]. There is evidence that gonadal hormones have an influence on the cannabinoid system in humans. For instance, research shows that endocannabinoid tone varies throughout the menstrual cycle. The highest levels of plasma anandamide are observed during ovulation, and they are positively associated with estradiol, luteinising hormone, and follicle stimulating hormone. These hormones may, therefore, play a role in regulating anandamide levels [59].

A single study conducted on CB1 receptor knockout animals showed that male CB1 receptor knockout mice displayed higher anxiety-like behaviour than wild-type controls, suggesting that CB1 receptors may function differently in males and females [60]. Moreover, a study of sex differences in brain endocannabinoid content in rats showed that females had higher 2-AG levels than males in the hypothalamus and pituitary but lower 2-AG levels in the cerebellum. Additionally, sex differences were found in the hippocampus (anandamide) and midbrain (2-AG and anandamide) based on the oestrous cycle. There is evidence that gonadal hormones have an influence on the cannabinoid system in humans. For instance, research shows that endocannabinoid tone varies throughout the menstrual cycle. The highest levels of plasma anandamide are observed during ovulation, and they are positively associated with estradiol, luteinizing hormone, and follicle stimulating hormone. These hormones may, therefore, play a role in regulating anandamide levels [36,59,61].

The first stage of our analysis was an association analysis of the number of AAT repeats and AUD. The study of the (AAT)n repeat number polymorphism in the *CNR1* gene revealed that in the group of alcohol use disorder subjects, significantly fewer repeats were present when compared with controls (12.04 vs. 12.40, *p* = 0.0380). On the other hand, Spanish alcoholic men (n = 107) who suffered from ADHD in childhood were found to have longer forms of *CNR1* alleles (more than 11 AAT repeats) [62]. A pioneering study by Comings et al. (1997) described the significant association between numbers of (AAT)n repeats and different drug (cocaine, amphetamine, and cannabis) dependence in non-Hispanic Caucasians (a mixed gender group), but it has not been observed in subjects with AUD. They showed that the alleles ≥ 14 repeats of AAT were associated with drug dependence [63]. In contrast, in the Chinese population, a significant association between (AAT)n polymorphism and heroin dependence was not found [64]. In a male Afro-Caribbean population, the frequency of the (AAT)12 repeat allele was higher in non-schizophrenic cocaine dependents and schizophrenic cocaine dependents [65]. The research showed that in both European-American and African American subjects, there were no significant differences in the frequencies of *CNR1* (AAT)n repeats observed between drug- or alcohol-dependent subjects and controls [66]. Unfortunately, these studies were focused on the association of (AAT)n polymorphism with substance dependence but did not examine the correlation of this polymorphism with personality traits. So far, there is very little research on this polymorphism in people with alcohol dependence, and in the available literature, we did not find any publication in which studies of this polymorphism in a group of women with AUD were described.

### 3.2. Personality Traits

Research has shown significant differences in personality traits between males and females. These differences have been attributed to both biological and socio-psychological factors. Studies have revealed that differences in hormone levels and social roles can shape personality traits [9]. Moreover, research has indicated differences in the size of specific brain regions, such as the hippocampus and amygdala, between males and females. Females tend to have a larger hippocampus, responsible for estrogen synthesis, while males tend to have a larger amygdala, which has the highest concentration of androgens [67]. Therefore, it is important to consider population differences when studying personality traits. Each population has a unique history and may have adapted differently to social, environmental, and other factors. These adaptations may have resulted in differences in genetic makeup that affect the expression of personality traits [68].

Currently, only a small fraction of the underlying genetic factors responsible for personality traits and alcohol use disorder (AUD) have been identified. This phenomenon is known as the “missing heritability problem”. It is believed that the most unexplained variation in these traits is due to the complex interplay between genetic factors, environmental influences, and their interactions.

One of the major questions that has arisen from the GWAS studies on personality and addiction is what causes “missing heritability”. Despite conducting GWAS on a large scale, researchers have been unable to account for more than a fraction of trait variance. There could be several factors that may be contributing to the “missing heritability”.

In a GWAS, the following variants: copy number variants (CNVs), insertions/deletions, variable number of tandem repeats (VNTRs), and short tandem repeats (STRs) are not evaluated. However, they may contribute to phenotypic variability [69,70,71].

The human genome is incredibly complex, with countless variants that can affect individual traits. This includes a vast array of psychological traits, each of which may be influenced by every single variant. As a result, exploring the links between genes and observable psychological traits is a formidable challenge. Nevertheless, with the right tools and expertise, researchers can uncover crucial insights that could transform our understanding of human behaviour and genetics.

The second stage of our analysis was the analysis of anxiety measured as a state, trait, and personality according to the “Big Five”. Comparing the alcohol use disorder subjects and the controls, we observed significantly higher scores on the Anxiety trait and state scales and the NEO Five-Factor Inventory Neuroticism and Openness (insignificant after correction) scales. The AUD subjects obtained significantly lower results on the Extraversion, Agreeability and Conscientiousness scales compared to controls. The third stage of our analysis was the correlation and regression analysis. We found no significant Pearson’s linear correlation between the number of (AAT)n repeats in the *CNR1* gene and the STAI scales nor the NEO Five-Factor Inventory traits studied in the group of AUD subjects. In contrast, controls showed a positive correlation between the number of (AAT)n repeats and the Anxiety state scale (insignificant after correction) and a negative correlation with the NEO-FFI Openness scale. Similar results were obtained for the linear regression models, i.e., we found no evidence for the cases, but a significant model was established for the anxiety measured as a trait and openness in controls.

In this study, we examined the correlations between (ATT)n polymorphism in the *CNR1* gene and personality traits in women with AUD. Nowhere else has such research been conducted on a selectively selected group of women with AUD. In other studies, it was found that (ATT)n polymorphisms are correlated with impulsivity in Southwest California Indians (a mixed gender group) [72]. Impulsivity was significantly associated with (AAT)6 repeat allele *CNR1*. There are no other reports in the literature on (AAT)n polymorphism and personality traits.

To our knowledge, studies conducted by other teams have only been based on the analysis of (AAT)n polymorphism and addiction [62,63,64,66,73] and no one has conducted such studies in the context of AUD and personality traits.

Our results regarding the correlation in the AUD group give rise to a few considerations.

First, correlation does not imply causation. The number of STR repeats in the *CNR1* gene in correlation with anxiety and openness to experience in the control group does not mean that the STR repeats are strongly influencing these traits. It is plausible to assume that other factors are influencing both traits. Secondly, the fact that this correlation is not observed in the group with alcohol use disorder (AUD) could be due to several reasons: (1) It is possible that the *CNR1* gene functions differently in individuals with AUD. Substance use disorder can cause various physiological changes, and it is likely that these changes could affect the function of the *CNR1* gene or its interaction with other genes. (2) While our sample sizes are not small, it might still be too small to detect a correlation in the AUD group; (3) There could be other factors at play that are confounding the results, i.e., individuals with AUD might have other genetic variations, environmental factors, or lifestyle factors that are influencing the relationship between STR repeats and openness and anxiety; (4) correlations can appear in one sample but not in another simply due to statistical fluctuation. This is especially true if the correlation is weak, as in our case.

It is worth noting that the examination of personality traits has solely focused on SNP polymorphisms in the *CNR1* gene, with no attention given to microsatellite polymorphism (AAT)n. Therefore, it is imperative that further research delves into this area to broaden our understanding of personality traits.

Bornscheuer et al. show an association of SNP polymorphism in the *CNR1* gene with personality traits [74]. Their study’s genotyping results indicate a trend between higher extraversion, lower agreeableness, and neuroticism and the allele of rs2023239. Other studies showed epistasis of rs806379 with several SNPs impacting neuroticism and agreeableness. The next significant epistasis was observed between rs1535255 and rs2023239, impacting agreeableness. Moreover, rs7766029 shows a significant interaction between recent negative life events and depression scores [75]. Alexandrova et al. also showed that not only *CNR1* polymorphism rs776609 but also polymorphisms of GABRA2 (rs9291283), GABRA6 (rs3219151), and MAMDC1 (rs7151262) are associated with neuroticism [76]. Yao et al. conducted a similar study on a group of African Americans [77]. His results indicated a correlation between 14 different polymorphisms of the *CNR1* gene and personality traits. Most of the studied SNPs were correlated with more than one personality trait. The greatest number was associated with extraversion (13 out of 14 SNPs), and none of these SNPs was correlated with neuroticism in their results. With conscientiousness were correlated rs806369, rs806371, rs9353526, rs9362466, and rs218619. Moreover, these SNPs were associated with extraversion and some with agreeableness (rs9353526, 9362466, rs2180619). Polymorphism rs806368 is related to extraversion, openness, and agreeableness. In their result, rs806381 is associated only with openness, and rs9444584, rs806372, rs806371, rs806370, and rs806368 are related to openness and other personality traits [77].

### 3.3. Short Tandem Repeat

The AAT polymorphism in the *CNR1* gene is classified as a short tandem repeat (STR). However, the precise functional implications of this polymorphism are yet to be fully understood. Some researchers believe that it may contribute to the formation of Z-DNA, which could, in turn, affect gene expression [31]. It is still unclear whether the AAT microsatellite is responsible for modified *CNR1* expression or is simply in linkage disequilibrium with a functional SNP [30].

It is worth noting that humans have over 1 million known STR loci, some of which can give rise to severe genetic disorders upon expansion. As a result, it is crucial to continue conducting research and furthering our understanding of these loci to aid in the prevention of these debilitating illnesses [78].

Short Tandem Repeat (STR) loci are crucial in determining the risk of disease and influencing biology in numerous ways. The surrounding DNA undergoes significant structural and functional changes due to STR loci. They can lead to the formation of hairpin loops [79] that obstruct DNA synthesis and promote chromosome breakage [80,81]. Additionally, GC-rich repeats in promoter regions can form stable G4 quadruplexes that regulate transcriptional activity [82,83]. Moreover, some STR loci can act as cis-regulatory elements, significantly enhancing transcription factor binding affinity up to 70-fold [84].

Since the Human Genome Project was completed, a lot of WGS data have been generated. However, the genetic variation in the length and sequence of STR loci is not well understood at the population level due to difficulties in accurately capturing and quantifying these regions with standard short-read sequencing. As a result, we have a limited understanding of the biological impact of this important class of genetic variation [85,86].

Short Tandem Repeats (STRs) are a fantastic tool that can help scientists map genetic traits and identify associations between STR repeat length dosage and gene expression or higher-order phenotypes. These STR loci, known as eSTRs, are particularly useful in this regard. Thousands of tandem repeats, including STRs and Variable Number Tandem Repeats (VNTRs), are associated with the expression of their respective genes or traits of interest. These eSTRs are often found in intronic and intergenic regions close to regulatory sequences, such as transcriptional start sites (TSS), DNase I hypersensitive sites, and 5′ and 3′ untranslated regions. Moreover, in some cases, eSTRs have a greater effect size than the lead SNP associated with gene expression. This suggests that STR variation can contribute to a significant proportion of missing heritability and disease burden [87,88]. Some eSTRs are even thought to act as powerful epigenetic regulators that influence the DNA methylation levels of CpG islands [89]. In short, STRs are an essential tool that can help scientists unlock the mysteries of genetics and disease.

### 3.4. Summary

The main advantage of our study is its undoubted uniqueness in terms of not only the group analysed—AUD women—but also the rare STR polymorphism analysed. To the best of our knowledge, this is the second study of this type conducted in a group of alcohol-dependent individuals and the first conducted in a group of women. In addition, as addiction is both a multigene and a multifactorial disease, our analysis is further enriched by the analysis of anxiety and personality traits. Adding important data to understanding AUD and AUD personality in women. Also, showing a significant correlation between the analysed polymorphic variant and anxiety and Openness in non-dependent subjects.

Our study is not free from certain limitations, i.e., (1) The study group consisted of 187 female subjects, with 93 diagnosed with alcohol use disorder (AUD) and 94 controls. While this sample size provides valuable insights, larger cohorts would enhance statistical power and allow for more robust conclusions. (2) The study focused exclusively on women with AUD. Extrapolating findings to broader populations (e.g., men, different ethnicities) requires caution. Diversifying the sample to capture a more representative cross-section of individuals affected by AUD would be beneficial. (3) Our study employed a cross-sectional design, capturing data at a single point in time. Longitudinal studies would offer dynamic perspectives, tracking changes in personality traits and genetic markers over time. (4) Personality traits and AUD status were assessed using self-reported inventories. Self-reported data may be influenced by social desirability bias or subjective interpretation. (5) While we explored genotype-personality associations, environmental factors (e.g., upbringing, stressors) play a crucial role in AUD susceptibility. Future research should investigate gene-environment interactions comprehensively. (6) Our focus was on the (AAT)n microsatellite polymorphism within the *CNR1* gene. Other genetic variants and epigenetic modifications within the *CNR1* gene may contribute to AUD risk as well. Expanding the genetic scope would yield a more comprehensive understanding. (7) We did not directly examine neurobiological pathways linking (AAT)n repeats to personality traits and AUD. Neuroimaging studies or animal models could elucidate the underlying mechanisms. (8) Although our study included only women, sex-specific effects may exist. Investigating male cohorts separately would provide insights into gender-specific vulnerabilities. (9) Our study is the first one analysing the (AAT)n repeats within the *CNR1* gene. Replicating our findings in independent cohorts is crucial for validation.

## 4. Materials and Methods

### 4.1. Participants

The study group included 187 female subjects. Of these, 93 were diagnosed with alcohol use disorder (mean age = 46.18, SD = 10.86), and 94 were not dependent on any substance or behaviour (control group; mean age = 45.81, SD = 9.67). The study was previously approved by the Bioethics Committee of the District Medical Council in Zielona Góra (KB-07/72/2017). Written informed consent was obtained from all participants, and the study was conducted at the Department of Clinical and Molecular Biochemistry, Pomeranian Medical University in Szczecin. Individuals with AUD and the control group were assessed by psychiatrists using the Mini-International Neuropsychiatric Interview (MINI), and both groups completed the NEO-FFI and STAI questionnaires independently.

### 4.2. Psychometric Tests

Our study employed a cross-sectional design. The Mini-International Neuropsychiatric Interview is a structured diagnostic interview to evaluate psychiatric diagnoses according to DSM-IV and ICD-10 criteria.

The Personality Inventory (NEO-FFI Five-Factor Inventory, NEO-FFI) contains six components for each of the five traits: neuroticism (anxiety, hostility, depression, self-awareness, impulsivity, susceptibility to stress), extroversion (warmth, sociability, assertiveness, activity, emotion seeking, positive emotions), openness to experience (fantasy, aesthetics, feelings, actions, ideas, values), agreeableness (trust, straightforwardness, altruism, compliance, modesty, tenderness), and conscientiousness (competence, order, duty, striving for achievements, self-discipline, consideration) [90].

The State-Trait Anxiety Inventory (STAI) assesses anxiety, which can be defined as a continuous tendency to experience worry, stress, and discomfort, as well as anxiety states such as fear and temporary activation of the autonomic nervous system prompted by particular situations, as a trait [91].

The results of the NEO-FFI and STAI are reported as sten scores. The conversion of raw scores to sten scores was carried out by following the Polish standards for adults [92,93], where it was assumed that 1–2 corresponded to very low results, 3–4 corresponded to low results, 5–6 corresponded to average results, 7–8 corresponded to high results, and 9–10 corresponded to very high results.

### 4.3. Genotyping

Genomic DNA was isolated from blood leukocytes using the QIAapm^®^ DNA Mini Kit (Qiagen, Hilden, Germany); all samples were sufficient for amplification. Repeat length polymorphisms of microsatellite regions (AAT)n in the *CNR1* gene were identified with polymerase chain reaction (PCR) using specific pairs of primers:

5′-6FAM-ACTCCGTCTCAAAAACAACAAAA-3′ (forward) and
5′-CTGCCATTAAGGGAAAGAGGT-3′ (reverse)

PCR conditions: T_h_60 °C, 34 cycles. The length of the PCR products was evaluated with capillary electrophoresis, using an ABI PRISM^®^ 3130 Genetic Analyzer (Applied Biosystems, Foster City, CA, USA) with a GeneScan™ 500 LIZ™ size standard.

Random samples underwent sequencing using primers:

5′-GCTACTCGGGAGGCTGAACC-3′ (forward) and
5′-CACCCCTGGGCTGTAAAATAACCT-3′ (reverse) to validate the identification of polymorphisms with capillary electrophoresis (PCR conditions: T_h_65 °C, 38 cycles). Due to the discrepancies found in the literature regarding the number of AAT repetitions in this microsatellite, homozygous individuals for the different alleles were sequenced. Sequencing was performed with the ABI PRISM^®^ 3130 Genetic Analyzer, and data were analysed with the Sequencing Analysis v5.1 software (Applied Biosystems, Waltham, MA, USA). All primers were designed based on the sequence NC_000006.12 (Homo sapiens chromosome 6, GRCh38.p14 Primary Assembly) from the database of the National Center for Biotechnology Information.

### 4.4. Statistical Analysis

The (AAT)n repeats number polymorphism in the *CNR1* gene, and individual traits measured using the STAI and NEO Five-Factor Inventory questionnaires did not have a normal distribution (Shapiro–Wilk *p* < 0.05).

To analyse the distribution of the (AAT)n repeats number polymorphism in the *CNR1* gene in the alcohol use disorder and control subjects, the Mann–Whitney U test was used, with *p* < 0.05 considered statistically significant.

The personality traits and anxiety measures of alcohol use disorder subjects, as measured by the STAI and NEO Five-Factor Inventory, were compared with the control group using the Mann–Whitney U test.

The relationship between the (AAT)n repeats number polymorphism in the *CNR1* gene and the personality traits measured by the STAI and NEO Five-Factor Inventory was shown separately in both study groups using Pearson’s linear correlation.

An attempt was also made to determine regression linear models between the number of (AAT)n repeats in the *CNR1* gene and the STAI scales and NEO Five-Factor Inventory traits studied in the group of AUD subjects and the control group. The Bonferroni multiple comparisons correction was applied for these variables, and the accepted significance level was 0.0071 (0.05/7). All statistical analyses were performed using STATISTICA 13 (TIBCO Software, Inc., Palo Alto, CA, USA) and PQStat software (v. 1.8.2., Poznań, Poland).

## 5. Conclusions

Scientists have been exploring the endocannabinoid system as a target for new anti-anxiety drugs. These drugs are designed to increase the availability of AEA and 2-AG or inhibit the enzymes FAAH and MAGL [33]. We conducted a study investigating whether a specific genetic variation, i.e., microsatellite polymorphism (AAT)n in the *CNR1* gene, is linked to personality traits in women with AUD. Our hypothesis suggests that a higher number of AAT repeats in the 3′ region of the *CNR1* gene may lead to lower availability of the CB1 receptor for AEA and 2AG, which in turn contributes to the occurrence of anxiety. Our current research supports this hypothesis.

Surprisingly, we observed a lower number of (AAT)n repeats in the AUD sample, and there was no correlation of (AAT)n repeats with anxiety or personality measures in the AUD sample, but it was significant. Following the hypothesis, correlations were observed in the controls. Additionally, the anxiety and personality measures differ significantly between the controls and cases. The lower number of (AAT)n repeats in the *CNR1* gene among AUD subjects suggests a potential link between this polymorphism and AUD susceptibility. However, the lack of a significant correlation between (AAT)n repeat length and personality traits within the AUD group underscores the complexity of this relationship. The AUD sample’s personality profile aligns with most literature reports. While the (AAT)n repeat length does not directly correlate with personality traits in AUD subjects, controls reveal intriguing correlations.

Interestingly, our study provided data on two separate complex issues: the association of (AAT)n *CNR1* repeats in the AUD and anxiety as a state in non-alcohol dependent subjects. In conclusion, our study provided a plethora of valuable data for improving our understanding of alcohol use disorder and anxiety. It opened several future directions: (1) functional implications of the (AAT)n polymorphism within the *CNR1* gene. Modulation of the endocannabinoid signalling pathways relevant to AUD susceptibility; (2) exploration of gene-environment interactions. Environmental factors modifying the impact of (AAT)n repeats on personality traits and AUD risk; (3) Investigating neurobiological pathways linking (AAT)n repeats, personality traits, and AUD vulnerability; (4) Epigenetic modifications—possible DNA methylation patterns at the *CNR1* locus role in shaping personality and AUD outcomes.

## Figures and Tables

**Table 1:** Frequency of *CNR1* (AAT)n allele frequencies in alcohol use disorder (AUD) and control subjects.

Number of AAT Repeats	Amplicon Size (bp)	AUD Number of Alleles	Control Subjects Number of Alleles
7	248	3 (1.61%)	-
10	257	53 (28.49%)	46 (24.47%
11	260	9 (4.84%)	12 (6.38%)
12	263	25 (13.44%)	30 (15.96%)
13	266	43 (23.12%)	34 (18.09%)
14	269	48 (25.81%)	64 (34.04%)
15	272	4 (2.15%)	2 (1.06%)
16	275	1 (0.54%)	-

**Table 2:** The (AAT)n repeat number polymorphism in the *CNR1* gene in alcohol use disorder (AUD) and control subjects.

(AAT)n Alleles	M	SD	Z	*p*
AUD n = 93 (alleles = 186)	12.04	1.74	−2.080	0.0380 *
Control n = 94 (alleles = 188)	12.40	1.58		

*p*-value of statistical significance in t-Student test; n, number of subjects; M ± SD—mean ± standard deviation; *—differences which are statistically significant (*p* < 0.05).

**Table 3:** STAI and NEO Five-Factor Inventory sten results in alcohol use disorder (AUD) and control subjects.

STAI NEO-FFI	AUD n = 93	Controls n = 94	Z (*p*-Value)
STAI trait scale	7.34 ± 2.21	4.92 ± 2.37	6.560 (<0.00001 *)
STAI state scale	5.87 ± 2.51	4.43 ± 2.22	3.964 (0.0001 *#)
Neuroticism scale	7.11 ± 1.84	4.46 ± 2.16	7.556 (<0.00001 *#)
Extraversion scale	5.14 ± 2.24	6.72 ± 1.93	−4.689 (0.00003 *#)
Openness scale	5.16 ± 2.14	4.42 ± 1.65	2.262 (0.0237 *)
Agreeability scale	3.83 ± 1.83	5.52 ± 2.22	−5.173 (<0.00001 *#)
Conscientiousness scale	4.88 ± 2.31	7.05 ± 1.99	−6.185 (<0.00001 *#)

*p*-value of statistical significance with Mann–Whitney U-test; n—number of subjects; M ± SD—mean ± standard deviation; *—differences which are statistically significant (*p* < 0.05). # Bonferroni correction was used, and the *p*-value was reduced to 0.0071 (*p* = 0.05/7 (number of statistical tests conducted)).

**Table 4:** Pearson’s linear correlation between the number of (AAT)n repeats in the *CNR1* gene and the STAI and NEO Five-Factor Inventory traits in alcohol use disorder (AUD) and control subjects.

	STAI Trait Scale	STAI State Scale	Neuroticism Scale	Extraversion Scale	Openness Scale	Agreeability Scale	Conscientiousness Scale
Number of (AAT)n repeats in the *CNR1* gene in AUD subjects	r = 0.012 (*p* = 0.875)	r= 0.000 (*p* = 1.00)	r= −0.064 (*p* = 0.385)	r = 0.051 (*p* = 0.488)	r= 0.106 (*p* = 0.149)	r = 0. 019 (*p* = 0.793)	r = 0.028 (*p* = 0.701)
Number of (AAT)n repeats in the *CNR1* gene in Controls	r = 0.184 (*p* = 0.011 *)	r= 0.057 (*p* = 0. 431)	r = 0.127 (*p* = 0.082)	r = −0.100 (*p* = 0.171)	r = −0.241 (*p* < 0.0001 *#)	r = −0.038 (*p* = 0.609)	r = 0.048 (*p* = 0.514)

r—Pearson’s linear correlation; *p*-value of statistical significance, *—differences which are statistically significant (*p* < 0.05). # Bonferroni correction was used, and the *p*-value was reduced to 0.0071 (*p* = 0.05/7 (number of statistical tests conducted)).

## Data Availability

The genotyping and psychometric tests’ results are available upon request.
